# Migration and primary healthcare in sub-Saharan Africa: A scoping review

**DOI:** 10.4102/phcfm.v16i1.4507

**Published:** 2024-07-12

**Authors:** Christian Lueme Lokotola, Robert Mash, Vincent Sethlare, Jacob Shabani, Ilori Temitope, Laurel Baldwin-Ragaven

**Affiliations:** 1 Department of Family and Emergency Medicine, Division of Family Medicine and Primary Care, Faculty of Medicine and Health Sciences, Stellenbosch University, Cape Town, South Africa; 2 Department of Family Medicine and Public Health Medicine, University of Botswana, Gaberone, Botswana; 3 Department of Family Medicine, Aga Khan University, Nairobi, Kenya; 4 Department of Family Medicine Unit, Faculty of Community Medicine, University of Ibadan, Ibadan, Nigeria; 5 Division of Family Medicine, Department of Family Medicine and Primary Care, School of Clinical Medicine, Faculty of Health Sciences, University of the Witwatersrand, Johannesburg, South Africa

**Keywords:** migration, internally displaced people, primary healthcare, primary care, Africa

## Abstract

**Background:**

Migration in Africa is increasing and driven by a variety of inter-related socio-economic, conflict and climate-related causes. Primary healthcare (PHC) migration on PHC service will be in the forefront of responding to the associated health issues.

**Aim:**

This study aimed to review the literature on the effect of migration on PHC service delivery in Africa and the challenges facing migrants in accessing PHC.

**Method:**

A systematic approach (Preferred Reporting Items for Systematic Reviews and Meta-analysis extension for Scoping Reviews) was applied across six databases and grey literature from African universities (2010 to 2021). Data were extracted and analysed quantitatively and qualitatively.

**Results:**

A total of 3628 studies were identified and 50 were included. Most studies were descriptive or used mixed methods. Publications came from 25 countries, with 52% of studies from South Africa, Uganda and Kenya. Most migrants originated from Zimbabwe, the Democratic Republic of Congo and Somalia. Population health management for migrant communities was challenging. Migration impacted PHC services through an increase in infectious diseases, mental health disorders, reproductive health issues and malnutrition. Primary healthcare services were poorly prepared for handling displaced populations in disaster situations. Access to PHC services was compromised by factors related to migrants, health services and healthcare workers.

**Conclusion:**

Several countries in Africa need to better prepare their PHC services and providers to handle the increasing number of migrants in the African context.

**Contribution:**

The review points to the need for a focus on policy, reducing barriers to access and upskilling primary care providers to handle diversity and complexity.

## Introduction

The migration and displacement of people from Africa are often in the news, particularly the plight of those trying to cross the Mediterranean to enter Europe for ‘a better life’.^1^ Although the media give the impression that migration is from countries in Africa to countries outside of Africa, the evidence is that most migration is within and between countries on the continent.^2^ In particular, sub-Saharan Africa is host to ‘more than 26% (over 18 million) of the world’s refugees’.^3^ Since 2010, migration within Africa has increased by 43.6%.^2^ Populations may be displaced because of conflict, with recent examples being the civil wars in Ethiopia and Sudan.^4^ Many people relocate for better opportunities and income, for example, moving from Zimbabwe or Malawi to South Africa, sometimes even for better healthcare or services.^1,5^ There are also examples of people being trafficked with promises of employment in other countries, for example, to the United Arab Emirates.^6^

A newly invigorated driver of migration on the African continent is climate change and the broader environmental crisis.^7^ The United Nations Intergovernmental Panel on Climate Change (IPCC) estimates that ‘over 2.6 million and 3.4 million new weather-related displacements occurred in sub-Saharan Africa in 2018 and 2019’.^8^ Looking to the future, they predict that ‘with 1.7 °C global warming by 2050, 17–40 million people could migrate internally in sub-Saharan Africa’.^9^

Primary healthcare (PHC) services are the most accessible in any health system. Outside of disaster situations, they are the first services to encounter migrant-related health issues. In Africa, PHC services are often poorly developed with inadequate infrastructure, unreliable supply chains and availability of medication, paper-based information systems and scarce human resources with inadequate training to function as generalists.^10^ Nevertheless, these healthcare systems face a future in which PHC will have to adapt to the increasing presence of migrants.^11,12^

Health services are often ineffective at responding to the needs of migrants, particularly when they are foreigners with different languages, culture and unofficial or marginalised status.^13,14^ Upon arrival in host countries, migrants with health-related problems have difficulties accessing PHC services. They are particularly vulnerable to abuse and exploitation.^13^ Many migrants have uncertain or illegal status in the country, which complicates and hinders access to services.^14^ Xenophobia is a reality in many countries, such as South Africa, and may affect the attitudes of healthcare workers.^13^

Médecins Sans Frontières (MSF) has called for a humane immigration and asylum system that protects the lives and health of people forced to leave their homes.^14^ The African Union report on migration and health underlined the potential health implications for migrants’ access to healthcare.^15^ The Lancet Commission on unmet health needs of refugees and migrants has shown multiple instances of discrimination in accessing services.^16^ As the African Charter on Human and People’s Rights states, ‘every individual shall have the right to enjoy the best attainable state of physical and mental health’, an entitlement that is not dependent on migration status.^17^ This is aligned with the World Health Organization’s (WHO) position on the health of migrants as a human right.^17^

This mismatch between the realities of health systems constraints and the burgeoning needs of migrant populations across Africa becomes a challenge to the PHC principles of ‘health for all’ and health equity. As we look to a future that is characterised by increasing migration, there is a need to understand the existing evidence in Africa on PHC and migration and to identify key research questions, which could help to build more resilient services in the future.

## Methods

### Study design

This was a scoping review of both published and unpublished scientific literature from the African continent. The scoping review followed a structured approach and was guided by the Preferred Reporting Items for Systematic reviews and Meta-Analysis extension for Scoping reviews (PRISMA ScR).^18^

### Search questions

The scoping review addressed the following two questions:

- What is the effect of migration on PHC service delivery in Africa? Service delivery includes the model and quality of care, resilience of facilities and services, as well as changes in morbidity or health problems, as a result of migration.
- What are the challenges facing migrants in accessing quality PHC services? Access would include issues of topography, acceptability of services, financial barriers, timeliness, language, or administrative barriers.

### Search strategy

#### Eligibility criteria

Table 1 lists the inclusion and exclusion criteria for the selection of articles.

**TABLE 1:** Inclusion and exclusion criteria for selection of articles.

Criteria	Include	Exclude
Population	Both migrants and host countries from within the African continent. Evidence from Africa settings.	Evidence from outside Africa.
Concept	Evidence addresses both migration and PHC in Africa. Any form of migration was included, both internal and external to a country, and for any reason.	Evidence only addresses one or none of these concepts.
Outcome	Evidence includes outcomes related to the effects of migration on PHC service delivery and migrants’ access to PHC services.	Evidence does not include these outcomes.
Study design	Original research studies that were quantitative, qualitative, mixed methods or research synthesis.	Not original research articles, such as reports, commentaries, editorials, opinion articles.
Time period	2010–2021	Outside this period.
Languages	English or French	Other languages.

PHC, primary healthcare.

#### Information sources

To identify the original studies and the grey literature related to PHC and migration, we systematically searched six databases: Scopus, PubMed, Cumulative Index to Nursing and Allied Health Literature (CINAHL), Web of Science, African Journals Online and Embase. The databases were chosen for their international coverage of health sciences literature, interdisciplinarity and complementarity. Cumulative Index to Nursing and Allied Health Literature specifically included publications in French. Research repositories from selected African universities were searched to incorporate the regional grey literature. Two top ranking African universities from each African region (southern, western, eastern, central and northern) were selected.^19^ In addition, several migration related organisational websites were searched for relevant research. These included: African Centre on Migration Studies, WHO, United Nations High Commissioner for Refugees (UNHCR), International Organization for Migration (IOM), United Nations, (International) Children (Emergency) Funds (UNICEF, UNCF), Medecins Sans Frontieres (Doctors without borders, MSF), African Union (AU) and Southern African Development Community (SADC).

#### Search terms

The search terms included both Medical Subject Headings (MeSH) and keywords to identify research studies that addressed the research questions (Table 2).

**TABLE 2:** Search terms.

Migration	Primary Health Care	Access
Migration	Primary health care	Access
Migrant*	Primary health care	Access*
Immigrant*	Primary care	Accessibility
Refugee*	District health services	Utili*ation
Internal migration	District health system	Availability
Internal displaced person		Acceptability
Asylum seeker		Afford*
Resettlement		Affordability
Displacement		Quality

Note: The asterisk shows the use of truncation and wildcards to search for different word variants or spellings.

Two electronic search strings were created (Box 1), each corresponding to one of the specific research questions. Each string was applied separately to each one of the information sources.

BOX 1 Search strings.

**Table:** 

**String 1:** (Migration OR migrant* OR immigrant* OR ‘internal migration’ OR refugee* OR resettlement OR ‘internal displaced person’ OR ‘asylum seeker’ OR displacement) AND (‘primary health care’ OR ‘primary healthcare’ OR ‘primary care’ OR ‘district health service’ OR ‘district health services’ OR ‘district health system’) AND (Africa OR Southern Africa OR East Africa OR Central Africa OR West Africa OR Sub-Saharan Africa OR Sub-Sahara Africa OR Sub Sahara Africa OR Angola OR Benin OR Botswana OR Burkina Faso OR Burundi OR Cameroon OR Cape Verde OR Central African Republic OR Chad OR Comoros OR Congo OR ‘Democratic Republic of Congo’ OR Cote d’Ivoire OR Ivory Coast OR Djibouti OR Equatorial OR Guinea OR Ethiopia OR Gabon OR Gambia OR Ghana OR Guinea OR Guinea-Bissau OR Kenya OR Lesotho OR Liberia OR Madagascar OR Malawi OR Mali OR Mauritania OR Mauritius OR Mozambique OR Namibia OR Niger OR Nigeria OR Rwanda OR ‘Sao Tome and Principe’ OR Senegal OR Seychelles OR Sierra Leone OR Somalia OR South Africa OR Sudan OR ‘South Sudan’ OR Somaliland OR Swaziland OR eSwatini OR Tanzania OR Togo OR Uganda OR Zambia OR Zimbabwe OR Morocco OR Algeria OR Libya OR Egypt OR Tunisia OR Western Sahara OR North Africa).
**String 2:** (Migration OR migrant* OR immigrant* OR ‘internal migration’ OR refugee* OR resettlement OR ‘internal displaced person’ OR ‘asylum seeker’OR displacement) AND (Access* OR accessibility, utili*ation OR availability OR acceptability OR afford* OR affordability OR quality) AND (Africa OR Southern Africa OR East Africa OR Central Africa OR West Africa OR Sub-Saharan Africa OR Sub-Sahara Africa OR Sub Sahara Africa OR Angola OR Benin OR Botswana OR Burkina Faso OR Burundi OR Cameroon OR Cape Verde OR Central African Republic OR Chad OR Comoros OR Congo OR ‘Democratic Republic of Congo’ OR Cote d’Ivoire OR Ivory Coast OR Djibouti OR Equatorial OR Guinea OR Ethiopia OR Gabon OR Gambia OR Ghana OR Guinea OR Guinea-Bissau OR Kenya OR Lesotho OR Liberia OR Madagascar OR Malawi OR Mali OR Mauritania OR Mauritius OR Mozambique OR Namibia OR Niger OR Nigeria OR Rwanda OR ‘Sao Tome and Principe’ OR Senegal OR Seychelles OR Sierra Leone OR Somalia OR South Africa OR Sudan ‘South Sudan’ OR Somaliland OR Swaziland OR eSwatini OR Tanzania OR Togo OR Uganda OR Zambia OR Zimbabwe OR Morocco OR Algeria OR Libya OR Egypt OR Tunisia OR Western Sahara OR North Africa).

### Selection process

Each member of the research team was allocated a particular information source to search with the above strings. As academics and researchers based in family medicine and PHC in Africa, each author conducted the initial search electronically on the assigned information source and screened the publications by title and abstract against the criteria. If there was doubt in terms of inclusion or exclusion, a second co-author was consulted. Duplicates were then removed and full text articles assessed against the eligibility criteria. Articles were not excluded based on quality of evidence because the objective of the scoping review was to map all the existing publications.

### Data collection process

Data from included publications were extracted independently by the author who was assigned to review that particular list of articles according to a standardised Excel template. The data extraction fields included: title; identifying features (first author, full citation, year of publication); study design features (study location, source country of migration, the research aim, the study design, the methods) and summary of the relevant findings related to our two main questions. Authors returned their extraction templates to C.L.L. who then integrated all the data into a single spreadsheet. The data were then interpreted by C.L.L. and R.M., and where necessary, the data were checked and clarified against the underlying article.

### Data analysis

The key characteristics of the studies were treated as categorical variables and reported as frequencies and percentages. The extracted results were analysed qualitatively using reflexive thematic analysis to identify the key themes that addressed the scoping review questions.^20^

## Review findings

### The search

**FIGURE 1:**
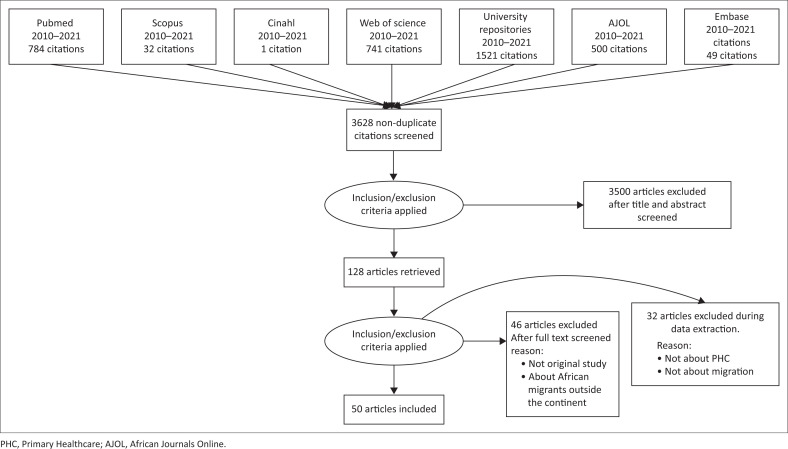
Flow diagram of selected studies.

The search identified 3628 research studies. After screening by title and abstract, 128 studies remained. A further 46 articles were excluded when reviewing the full text. After excluding 32 more articles during the data extraction process, 50 studies were included in the final review (Figure 1). The articles are summarised in Online Appendix 1.

### Characteristics of included studies

**FIGURE 2:**
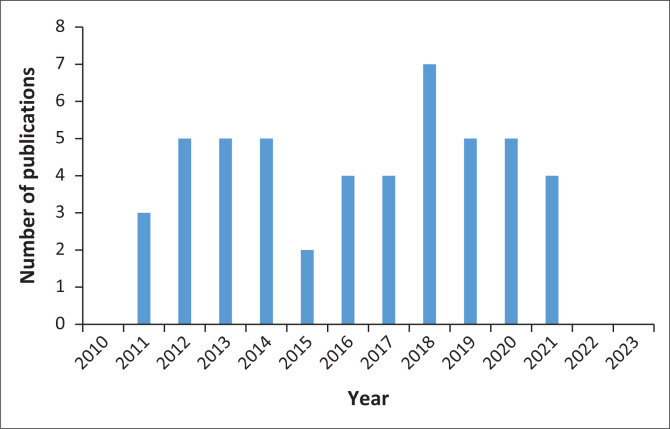
Number of publications per year.

Table 3 describes the characteristics of the selected studies. Out of the 25 countries where data were collected, 26 out of the 50 studies (52%) were conducted in South Africa, Uganda and Kenya. Migrants came from 32 different African countries, but research on those originating from Zimbabwe, Democratic Republic of Congo, Somalia, Ghana and Uganda predominated, with several studies including migrants from several different countries in the host country. The number of publications per year was consistently small and showed no particular trend over time (Figure 2). Most studies were descriptive or mixed methods.

**TABLE 3:** Characteristics of the studies.

Summary	Frequency
**Study location**	
South Africa	14
Kenya	7
Uganda	7
Nigeria	5
Ghana	5
Ethiopia	3
Rwanda	2
South Soudan	2
Burkina Faso	2
Tunisia	1
Lesotho	1
Mozambique	1
Mali	1
Burundi	1
Chad	1
Liberia	1
Zambia	1
Namibia	1
Djibouti	1
Tanzania	1
Republic of Central Africa	1
Malawi	1
Democratic Republic (DR) of Congo	1
**Migrants’ countries of origin**	
Zimbabwe	13
DR Congo	9
Somalia	8
Ghana	7
Uganda	6
Malawi	5
Mozambique	5
Chad	5
Cote d’Ivoire	4
Kenya	4
Ethiopia	4
South Soudan	4
Rwanda	4
Tanzania	4
Burundi	3
Niger	3
South Africa	2
Cameroon	2
Sudan	2
Zambia	2
Mali	2
Djibouti	2
Liberia	2
Nigeria	2
Namibia	1
Lesotho	1
Swaziland	1
Libya	1
Togo	1
Burkina Faso	1
**Study design of included studies**	
Descriptive cross-sectional survey	17
Mixed methods	11
Secondary analysis	8
Exploratory descriptive qualitative study	6
Reviews	4
Case study	3
Ethnographic study	1

The key themes derived from the data and answering the two review questions are presented next.

### Increase in mortality and morbidity among migrants

Many migrants and internally displaced people experienced health hazards and increased morbidity placing strain on the healthcare system.^21,22^ Mortality rates are often high among migrants and internally displaced populations.^23^ Increased incidence of polio, psychological and mental health disorders such as depression in Borno State; Nigeria was reported among internally displaced people from Dikwa and Kala Balge, exacerbating already strained resources for the local population.^24,25^ The increased health hazards were because of poor environmental conditions, poor infrastructure, a lack of income to pay for services and reliance on poor quality, often informal and unregulated health providers.^26,27,28^ The lack of income and informal status encouraged transactional sex, with risks of human immunodeficiency virus (HIV) and sexually transmitted infections.^29,30^ During transit to host countries, migrants were often vulnerable to sexual assault and abuse, predisposing them to HIV, sexually transmitted infections and unwanted pregnancies, as well as psychological trauma.^29,31^

Typical health problems among migrants included pneumonia, malaria, upper respiratory tract infections (URTI), diarrhoea, measles, malnutrition and mental health disorders such as depression and anxiety.^32,33^ The traumatic experiences that refugees undergo during migration may result in anxiety and post-traumatic stress disorders.^34^ Depression and psychosis can lead to hospital admissions and be associated with suicide and substance abuse.^35^ The lack of integrated primary mental healthcare is particularly problematic given the high rates of mental health problems.

### Problems with providing primary healthcare in humanitarian settings

African PHC systems suffer from a lack of financing and poor governance, with an over reliance on international donor funds to provide health services for migrants and refugees.^36^ Healthcare for internally displaced people may be challenging in such contexts and suffer from poor coordination between the national level and implementing agencies.^37^ When displacement or migration was associated with conflict or other crises there might also be destruction of health infrastructure and a lack of health system inputs such as essential equipment, supplies and medication.^38^ Service delivery in temporary or refugee camps was often not comprehensive, poorly integrated and insufficient.^39^ Camps for internally displaced people may lack supplies of medication and human resources for health and inadvertently encourage people to seek help from alternative and informal healthcare providers.^38^ Lessons learned from helping babies survive in humanitarian settings in Cameroon, Somalia, Niger and Chad highlighted the limited financial resources, a lack of regular training and insufficient capacity for essential newborn care.^40^ There is often limited time for healthcare providers to consult with people living in camps and to address key issues such as maternal and child health, as shown in Uganda, Cameroon and Chad.^11,31^

### Difficulties with community-orientated primary care

Although the influx of people puts a strain on PHC planning and service delivery in host countries, data on actual numbers, types of migrants and their demographics are often not available or unreliable.^41^ It is often difficult for public health authorities to define and engage with the migrant communities because of a lack of clear health data.^42,43^ At the same time, there is a major discrepancy between the very low rates of service utilisation compared to high rates for post-traumatic stress disorder, anxiety and depression.^44^ Proactive outreach is difficult, especially for vulnerable sub-groups such as children and adolescents.^45,46^ For example, childhood immunisation coverage may be low in-migrant populations and thus drive the continued spread of polio.^47^

### Confusing or discriminatory health policy

National policies on health services for migrants might exclude migrants from essential services or might be contradictory and difficult to interpret.^36,46,48^ Some were not eligible for social services or social protection.^49^ The impression was that the need to regulate and contain migrants could outweigh other rights and protections, for example, for pregnant and lactating women or children.^47,50^

### Migrant-related factors that impede access to primary care services

Migrants were often on the margins of communities and fearful of xenophobic attacks and other forms of social exclusion.^48,49,50^ When migrants were isolated, poorly integrated into communities and lacked social support, their ability to access health services was impaired.^51^ Migrants might be involved in exploitative labour practices that made it difficult to attend health services for fear of losing one’s job.^52^ For some, obtaining an income would be more important than accessing healthcare or protecting oneself from HIV and sexually transmitted infections in sex work.^53^ Illegal immigrants were also afraid of arrest and deportation if they contacted formal government health services and were registered there.^44,54^ As a result, migrants often used informal or unqualified health services such as traditional birth attendants (TBA), drug peddlers, herbalists or pharmacies.^55,56^ Home deliveries with help from TBAs might be common.^57^ In Ghana, many of the female porters (so called ‘*Kayayei*’) sought healthcare from alternative sources such as drug peddlers, herbalists and chemists, circumventing formal PHC services.^58^ Such behaviour can lead to late diagnosis of medical problems, such as HIV/AIDS, as well as reduced effectiveness of treatment.^30^ Migrants might also have a strong belief in the use of traditional practitioners and medications.^44,45^

### Health service-related factors that impede access to primary care services

African PHC services often lack comprehensiveness and quality of care, and these weaknesses are compounded by difficulty providing services for migrants.^59,60,61,62,63^ Migrants may struggle to obtain information about how the health services were organised or what was available.^51,52^ They may not know what their rights were in healthcare.^53^ They may also feel local services are linguistically or culturally unacceptable or too unfamiliar to feel comfortable.^54^ Language barriers were a major problem for many migrants and sometimes for the healthcare providers as well and the use of informal translators can also create problems.^40,41,55,56^ Sharing a similar culture and language between neighbouring countries can help overcome such fears.^57^

Cost can also be a major barrier.^48,53,54,64,65^ Healthcare workers may charge increased amounts that were not part of policy, thus contributing to discrimination.^48^ Migrants may not understand how the costs works or how insurance is provided. A study on health insurance of migrants in Kenya found that insurance cover is linked to older age (more income and resources), education, no religion, being married, having access to information and poorer language proficiency.^48^

A lack of access to reproductive health services (antenatal care, family planning) was highlighted in many studies.^39,56,61,63,66,67,68^ Low utilisation of family planning raises the risk of unplanned pregnancies.^69^ For some, this also increased the risk of birth complications because of female genital cutting.^55^ In conflict zones, there was a fear of reporting rape and sexual violence.^38,70^

There were conflicting reports on access to antiretroviral therapy for HIV. Migrants were able to continue accessing antiretroviral therapy (ART) in their home country and then import them into their host country. Migrants in Burkina Faso could access highly active antiretroviral treatment, while in South Africa reports were conflicting.^30^ One study suggested that cross-border migrants were successful in accessing and continuing ART in South Africa.^51^ Another study of a South African tertiary hospital found no difference in ease of access between migrant and non-migrant children and no difference in prior PHC attendance (antenatal attendance, delivery outcomes, immunisation cover and HIV prevention).^71^ Although another study, however, found dissatisfaction with antenatal care among migrants.^72^

### Health worker-related factors that impede access to primary care services

Migrants might be made to wait and put to the back of the queue when trying to access care.^73^ Health services might be unacceptable because of negative attitudes from healthcare workers, ranging from carelessness to harassment and refusal of services.^64^

## A summary of key findings

Throughout the decade under review, there were very few studies per year exploring the phenomenon of PHC and migration in the African context, and most studies were descriptive or exploratory in design. Studies described a plethora of health issues faced by migrants with a particular emphasis on infectious, reproductive and sexual health issues as well as mental health disorders. Internally displaced communities in camps were particularly challenging as health systems were unprepared in terms of finances, equipment, supplies, human resources and training to deal with these situations. Mothers and children were especially vulnerable in humanitarian settings.

**FIGURE 3:**
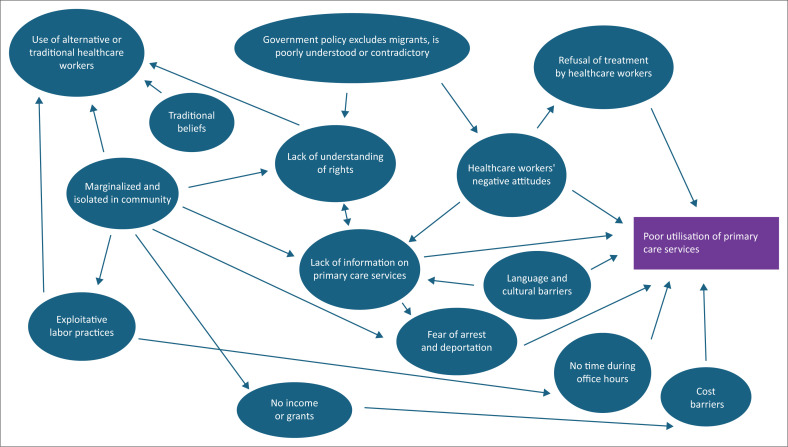
Factors impeding access to primary care services.

The complexity and interconnectedness of the issues that impede access to primary care services are summarised in Figure 3. Immediate barriers appear to be language and cultural differences, costs, negative attitudes and discrimination by healthcare workers, a lack of information about health services as well as fears of reporting, arrest and deportation. Underlying these immediate barriers are related factors such as a lack of understanding of their rights by both healthcare workers and migrants, poverty, exploitative labour practices, traditional beliefs on healthcare and being marginalised and isolated within local communities. The policy environment may also be hostile, contradictory or misunderstood.

### Discussion of key findings

Primary healthcare facilities and services appear unprepared to handle the needs and challenges of migrants and displaced populations.^61,65,74,75^ The predicted increase in the number of migrants, particularly with climate change, is likely to further challenge PHC services.^42,76,77^ Not only do migrants bring additional and sometimes less familiar health problems but can also act as vectors for infectious diseases.^24^ For example, the recent cyclone in southern Malawi led to an outbreak of cholera in South Africa as people migrated.^78^ Addressing the health needs of migrants can therefore also be protective of the receiving populations.^79,80^ The Lancet Commission on Migration and Health confirms that increased mental illness was found among migrants, refugees and internally displaced people in the country of destination.^77^

Migrants can also inflate the population served by a PHC facility and lead to unexpected increases in workload, which could contribute to greater burnout and job stress among healthcare workers.^81^ This is problematic in settings where PHC services are already under-resourced and under strain from long queues and waiting times. Discrimination by healthcare workers against migrants may be fuelled by the perception that they are increasing the workload.

Cultural and linguistic barriers to healthcare can be frustrating for both patients and healthcare workers.^81^ Migrants may struggle to speak the local languages and at the same time healthcare workers may not speak languages from other countries. Informal translators can be unreliable and create issues with confidentiality. Translation services may be available telephonically or online but still difficult to access during the consultation. Language barriers can reduce the utilization of the services. They can also add to discrimination as consultations take longer and are more difficult.

This review underlines the challenge to managing migrants’ health in the community. For example, the International Organization on Migration (IOM) has highlighted the difficulties with tuberculosis (TB) prevention, control and elimination strategies among migrants.^82^ According to The Lancet Commission on Migration and Health, a lack of data about the health of undocumented migrants challenges PHC capacity and assistance.^77^ A community-orientated primary care approach has equity as a key principle and this implies the need to look at healthcare and health needs among the most vulnerable, for instance, vulnerable migrants.^83^ Inequity is fuelled by a myriad of interconnected issues as shown in Figure 3 such as lack of understanding of their rights and how primary care services work, exploitative labour practices, fears of arrest or deportation and poverty.

There is a need to respond to migration in the planning of PHC services in Africa and the building of greater resilience.^84^ Primary healthcare facilities and services need to be better prepared to respond to and continue functioning despite climate hazards and disaster situations. This has been demonstrated through cyclones in countries such as Nigeria, Mozambique and Malawi as well as in situations of extreme heat, drought and water scarcity.

### Strengths and limitations

The current scoping review explored a number of international databases and several African universities data repositories over an 11-year period from 2010 to 2021. Despite this, it is possible that unpublished research studies in the African region could have been missed. Furthermore, although the search for evidence was conducted in both French and English, all 50 included articles were English language publications. As other languages are used across the African continent, this literature would have been excluded at the outset. All authors participated in the search and extraction of data and there might have been some individual variation in the application of inclusion criteria and interpretation of study’s findings. Nevertheless, two authors (CLL and RM) interpreted the data, and where necessary checked the extracted data against the original full text article.

### Implications and recommendations

This review underscores that PHC services and facilities on the African continent appear unprepared to handle the diverse health needs of migrants and displaced populations. At the same time, the number of people on the move is likely to increase. These findings therefore have serious implications, for which we propose the following recommendations:

- Ensure that national policies on migrants’ rights and the responsibilities of PHC services are clear, aligned with regional and international norms and communicated to PHC providers.
- Make sure to implement such policies so that population health management and community-orientated primary care gives attention to the needs of migrant populations living within the communities served. This helps to break isolation because of lack of knowledge.
- Improve access to PHC services for migrants, specifically by removing unnecessary barriers to care such as, unfair fees and addressing stigma, discriminatory and xenophobic attitudes among healthcare providers.
- Train primary care providers in the management of unusual diseases or outbreaks as a result of migration to meet the diverse health needs of migrants.
- Train primary care providers in their roles and competencies required to assist with disaster situations and displaced populations.

## Conclusion

Migrants throughout Africa challenge PHC services in host African countries with an array of health issues, particularly infectious diseases, mental health disorders, sexually transmitted infections, unwanted pregnancies and malnutrition. Migrant communities are difficult to include in community-orientated primary care, as they are marginalised and hidden. Infectious diseases may also pose risks to the community as a whole. Primary healthcare is unprepared to cope with displaced populations in disaster situations, and many facilities and services lack resilience. Maternal and child health is a particular challenge in these situations. Access to PHC is limited by a wide range of factors, such as exploitative labour practices and the fears of migrants to approach government facilities. Traditional beliefs, cultural and language barriers may be important. A lack of information on facilities and services as well as financial barriers also contribute. Policy on migrants may be confusing and allow primary care providers to adopt discriminatory practices. Primary care facilities, services and providers need to be better prepared to deal with the anticipated increase in migration over the coming decade throughout Africa.
